# High-Dose Exposure to Polymer-Coated Iron Oxide Nanoparticles Elicits Autophagy-Dependent Ferroptosis in Susceptible Cancer Cells

**DOI:** 10.3390/nano13111719

**Published:** 2023-05-24

**Authors:** Thanpisit Lomphithak, Selin Helvacioglu, Ilaria Armenia, Sandeep Keshavan, Jesús G. Ovejero, Giovanni Baldi, Costanza Ravagli, Valeria Grazú, Bengt Fadeel

**Affiliations:** 1 Division of Molecular Toxicology, Institute of Environmental Medicine, Karolinska Institutet, 17177 Stockholm, Swedensandeep.keshavan@unifr.ch (S.K.); 2 Department of Clinical Chemistry, Faculty of Allied Health Sciences, Chulalongkorn University, Bangkok 10330, Thailand; 3 Department of Molecular Biology and Genetics, Izmir Institute of Technology, Izmir 35433, Turkey; 4 Instituto de Nanociencia y Materiales de Aragón (INMA), CSIC-Universidad de Zaragoza, 50001 Zaragoza, Spain; 5 Instituto de Ciencia de Materiales de Madrid (ICMM-CSIC), 28049 Madrid, Spain; 6 Department of Dosimetry and Radioprotection, General University Hospital Gregorio Marañón, 28049 Madrid, Spain; 7 Colorobbia Consulting S.R.L., Sovigliana, 50053 Vinci, Italy; baldig@colorobbia.it (G.B.); ravaglic@colorobbia.it (C.R.); 8 Centro de Investigación Biomédica en Red de Bioingeniería, Biomateriales y Nanomedicina (CIBER-BBN), 50018 Zaragoza, Spain

**Keywords:** autophagy, cobalt, ferroptosis, iron oxide nanoparticles, lipid peroxidation

## Abstract

Ferroptosis, a form of iron-dependent, lipid peroxidation-driven cell death, has been extensively investigated in recent years, and several studies have suggested that the ferroptosis-inducing properties of iron-containing nanomaterials could be harnessed for cancer treatment. Here we evaluated the potential cytotoxicity of iron oxide nanoparticles, with and without cobalt functionalization (Fe_2_O_3_ and Fe_2_O_3_@Co-PEG), using an established, ferroptosis-sensitive fibrosarcoma cell line (HT1080) and a normal fibroblast cell line (BJ). In addition, we evaluated poly (ethylene glycol) (PEG)-poly(lactic-co-glycolic acid) (PLGA)-coated iron oxide nanoparticles (Fe_3_O_4_-PEG-PLGA). Our results showed that all the nanoparticles tested were essentially non-cytotoxic at concentrations up to 100 μg/mL. However, when the cells were exposed to higher concentrations (200–400 μg/mL), cell death with features of ferroptosis was observed, and this was more pronounced for the Co-functionalized nanoparticles. Furthermore, evidence was provided that the cell death triggered by the nanoparticles was autophagy-dependent. Taken together, the exposure to high concentrations of polymer-coated iron oxide nanoparticles triggers ferroptosis in susceptible human cancer cells.

## 1. Introduction

Iron-containing nanomaterials, including iron oxide nanoparticles, have been identified as promising nanomaterials for a host of biomedical applications, ranging from medical imaging to drug delivery or including a combination of both (aka theranostics) [1]. Needless to say, every nanomaterial envisioned for clinical applications must be thoroughly scrutinized with respect to its biocompatibility and biodistribution profiles [2].

In addition to being deployed as contrast agents (for magnetic resonance imaging) or as drug delivery vehicles, iron oxide nanoparticles have also been investigated for their intrinsic anti-cancer activities. For instance, iron oxide nanoparticles may be harnessed for magnetic hyperthermia in which the administration of magnetic nanoparticles is followed by the application of an alternating external magnetic field, thereby generating heat within the tumor area, with the subsequent necrosis of tumor cells taking place [1]. More recently, iron oxide nanoparticles have been suggested for use in triggering ferroptosis, a regulated form of necrosis that appears to be a particular vulnerability of cancer cells [3]. Ferroptosis is an iron-dependent, lipid peroxidation-mediated cell death, and it has been shown that therapy-resistant cancer cells remain sensitive to ferroptosis [4]. Notably, several studies have shown that iron oxide nanoparticles are capable of triggering ferroptosis in cancer cells (reviewed in: [5]). For instance, Fernández-Acosta [6] reported that iron oxide nanoparticles coated with gallic acid and polyacrylic acid triggered ferroptosis in a human fibrosarcoma cell line, while Bae et al. [7] reported that hyaluronic acid-functionalized iron oxide nanoparticles triggered ferroptosis in a human lung adenocarcinoma-bearing mouse xenograft model. Gao et al. [8] showed that so-called ultrasmall (10 nm) iron oxide nanoparticles triggered changes in intracellular iron metabolism in human breast cancer cells, leading to ferroptosis. Furthermore, Wen et al. [9] found that similarly-sized iron oxide nanoparticles triggered ferroptosis in a human glioblastoma cell line with the involvement of autophagy. However, it is notable that some authors have found that iron oxide nanoparticles can also trigger ferroptosis in normal (non-cancerous) cells, such as vascular endothelial cells [10]. Cobalt ferrite (CoFe_2_O_4_) nanoparticles are another widely investigated class of magnetic nanoparticles for which cytotoxicity has been observed in certain carcinoma cell lines [11,12] while only mild toxicity was recorded in human brain microvascular endothelial cells [13]. Indeed, it is important to consider that not all iron oxide nanoparticles trigger cell death, as exemplified by the use of iron-based nanoparticles as oral iron supplements in the management of anemia [14]. Therefore, further studies are needed to determine whether iron oxide nanoparticles trigger ferroptosis in normal versus cancerous cells. In the present study, we investigated iron oxide nanoparticles with and without cobalt functionalization (Fe_2_O_3_ and Fe_2_O_3_@Co-PEG) and PEG-PLGA-coated iron oxide nanoparticles (Fe_3_O_4_-PEG-PLGA), using a cancer-derived cell line versus a normal fibroblast cell line. Cell death (ferroptosis) was observed at high concentrations in susceptible cancer cells.

## 2. Materials and Methods

### 2.1. Nanoparticles

*Fe_2_O_3_ nanoparticles*: The synthesis of the Fe_2_O_3_ and Fe_2_O_3_@Co-PEG nanoparticles was carried out using a modified Massart co-precipitation protocol, as previously reported [15]. Briefly, a solution containing 0.2 M FeCl_3_·6H_2_O and 0.12 M FeCl_2_·4H_2_O was added to a mixture of 75 mL of NH_4_OH (25%), which was then heated to 90 °C for 1 h. The resulting mixture was cooled and washed with distilled water. To convert the particles into a single phase of maghemite (γ-Fe_2_O_3_), the suspension was treated with 2 M HNO_3_ for 15 min, followed by the addition of 1 M Fe(NO_3_)_3_ and water. The mixture was heated and stirred for an additional 30 min, then it was washed with acetone and redispersed in water. To stabilize the surface of the particles, a coating of dimercaptosuccinic acid (DMSA) was applied. Afterward, DMSA at 0.027 moles was dissolved in 5 mL of deionized water and mixed with 43 mg of the particles at pH 3 under stirring. The pH was then raised to 11 and sonicated for 20 min, followed by dialysis against deionized water for 48 h. Cobalt was introduced on the particle surface by functionalization with a chelating agent coordinated with divalent covalent cations (NTA-Co^2+^). To this end, a modified nitrilotriacetic acid (NTA), named Nα,Nα-Bis(carboxymethyl)-L-lysine hydrate, was complexed by coordination at 45 mM in a 20 mM HEPES buffer pH 8.0 with 50 mM of CoCl_2_. Then, the pH of the solution was adjusted to 10.5 using 30% (10 N) sodium hydroxide. The solution obtained was centrifuged at 4000× *g* for 10 min to eliminate the non-complexed cobalt ions. The supernatants were collected and used for nanoparticle functionalization following the adjustment of the pH to 8.5–8.9. A solution of 1-(3-dimethylaminopropyl)-3-ethylcarbodiimide (EDC) and *N*-hydroxysulfosuccinimide (NHS) (both from Sigma Aldrich, St. Louis, MI, USA) was prepared and added to 1 mg Fe of the nanoparticles in the 1 mL final volume. The suspension was then incubated with 500 μL of NTA-Co^2+^ for 2 h at room temperature. To increase the colloidal stability of the Fe_2_O_3_@Co-PEG nanoparticles, 100 μL of a 4 μM solution of α-methoxy-ω-amino PEG of 5000 Da (RappPolymer, Tübingen, Germany) was added and stirred for 45 min. The particles were separated by magnet or centrifugation and suspended in fresh buffer before being stored at 4 °C. The amount of cobalt incorporated into the particle surface was quantified by ICP (inductively coupled plasma) spectroscopy and found to be 0.2% of the total sample [15]. Transmission electron microscopy (TEM) of both particles was performed by depositing the particles onto copper grids with porous carbon films followed by an examination using a TECNAI G2 microscope operating at 200 kV. Prior to deposition, the particles were sonicated for 5 min in Milli-Q^®^ water. Dynamic light scattering (DLS) analyses of the particles dispersed in Milli-Q^®^ water and in cell culture medium were performed using a Malvern Zetasizer Nano ZS [15]. *Fe_3_O_4_ nanoparticles*: The synthesis of the Fe_3_O_4_-PEG-PLGA nanoparticles was performed as previously described [16]. Briefly, 40 g of magnetite (Fe_3_O_4_—1.1% *w*/*w* in Fe_3_O_4_) suspended in diethylene glycol was sonicated for 1 h in an ultrasound bath, and then a solution of [*N*-(3,4-dihydroxyphenethyl)dodecanamide (DDA)] (1.09 g, 3.2 mmol) dissolved in 120 mL of ethanol was slowly added to the glycolic suspension. The system was gently mixed for 1 h at room temperature. The solution was then diluted with 60 mL of ultrapure water, magnetically sedimented, and dispersed in 90 mL of THF (Fe_3_O_4_–DDA) (0.92 g, 2.7 mmol). Then, 440 mg of PGLA-*b*-PEG-COOH (43–3 kDa, 9.6 μmmol) were dissolved in 10 mL of THF and added to 90 mL of Fe_3_O_4_–DDA suspension (440 mg of Fe_3_O_4_). The formation of Fe_3_O_4_-PEG-PLGA was achieved by using the nanoprecipitation method [17]. To this end, the organic phase was mixed with 1000 mL of ultrapure water under vigorous stirring, maintaining a water-to-organic ratio of 10:1 with the constant removal of the resulting solution. The mixture was then purified by washing with a phosphate-buffered solution and concentrated (Pellicon XL, 500 kDa, Merck, Darmstadt, Germany) to a final volume of 150 mL [0.3% (*w*/*w*) in Fe_3_O_4_]; the mixture was then filtered through a polyethersulfone membrane (0.22 μm, Millipore Sterivex™). The TEM and DLS analyses of Fe_3_O_4_-PEG-PLGA nanoparticles were reported previously by us [18].

### 2.2. Human Cell Lines

The human fibrosarcoma cell line (HT1080) and normal human fibroblast cells (BJ) were obtained from American Type Culture Collection (ATCC). The cells were grown in the minimum essential medium (MEM) (Gibco, Waltham, MA, USA) supplemented with 10% fetal bovine serum (FBS) (Gibco) and 100 μg/mL streptomycin (Gibco). The human acute monocytic leukemia cell line THP-1 and human acute promyelocytic leukemia cell line HL-60 were also purchased from ATCC. The THP-1 cells were grown in a RPMI-1640 medium with GlutaMAX™ containing HEPES (Gibco) and supplemented with 100 U/mL penicillin, 100 mg/mL streptomycin, and 10% heat-inactivated FBS (Sigma). For differentiation into macrophage-like cells [19], THP-1 cells were plated at a density of 60.000 cells/well and incubated for 24 h in the presence of 0.5 μM PMA. HL-60 cells were maintained in a phenol red-free RPMI-1640 medium supplemented with 2 mM l-glutamine and 10% heat-inactivated FBS (Sigma). To allow for differentiation into neutrophil-like cells [19], the cells were seeded at 0.5 × 10^6^ cells/mL in a cell medium supplemented with 1.25% DMSO for 5 days, changing the medium after 3 days. The cell lines were all tested regularly using the MycoAlert^®^ mycoplasma detection kit (Lonza, Basel, Switzerland).

### 2.3. TEM Analysis

TEM was performed to visualize the uptake of particles. The HT1080 and BJ cells were exposed to 50 μg/mL of Fe_2_O_3_ or Fe_2_O_3_@Co-PEG nanoparticles for 24 h. The cells were then harvested and fixed in 2.5% glutaraldehyde in 0.1 M phosphate buffer (pH 7.4) at room temperature for 30 min and further fixed overnight in the refrigerator. After washing with 0.1 M phosphate buffer, the samples were centrifuged and post-fixed in 2% osmium tetroxide in 0.1 M phosphate buffer (pH 7.4) at 4 °C for 2 h. The samples were dehydrated in ethanol and acetone before being embedded in LX-112. Ultrathin sections (50–60 nm) were cut using a Leica EM UC6 microtome and being contrasted with uranyl acetate and lead citrate; the samples were examined using a Tecnai™ 12 Spirit Bio TWIN TEM at 100 kV. Digital images were captured using a Veleta camera (Olympus, Tokyo, Japan).

### 2.4. ICP-MS Analysis

Inductively coupled plasma mass spectrometry (ICP-MS) was performed to quantify the cellular uptake of Fe_2_O_3_ and Fe_2_O_3_@Co-PEG nanoparticles in the HT1080 and BJ cells. The cells were seeded in 6-well plates, exposed to 50 μg/mL of the nanoparticles for 24 h, and then washed with PBS and harvested for the analysis of Fe and Co content, as described [20]. The samples were digested with 65% nitric acid for 48 h and diluted with 2% nitric acid prior to loading onto the iCAP™ RQ ICP-MS operator (Thermo Fisher Scientific, Waltham, MA, USA). The calibration standards for Fe and Co were prepared using the single element standard, and all samples were spiked with 5 ppb indium as an internal standard. The Fe and Co concentrations were calculated using the standard curves.

### 2.5. LDH Release Assay

The lactate dehydrogenase (LDH) release assay was applied to assess the cytotoxicity of the various nanoparticles by measuring the level of LDH leakage into the medium. The CytoTox 96^®^ Non-Radioactive Cytotoxicity Assay Kit (Promega, Madison, WI, USA) was used according to the manufacturer’s instructions. The different cell lines were seeded at 5000 cells/well and were then exposed to Fe_2_O_3_ or Fe_2_O_3_@Co-PEG nanoparticles at concentrations ranging from 10 to 400 μg/mL, or to Fe_3_O_4_-PEG-PLGA nanoparticles at concentrations ranging from 5 to 100 μg/mL (higher concentrations could not be achieved). To evaluate the potential mechanism of cytotoxicity, the cells were preincubated for 30 min with either the pan-caspase inhibitor zVAD-fmk (20 μM), the iron-chelating agent deferoxamine (DFO) (10 μM), the soluble vitamin E analog Trolox (500 μM), the radical trapping antioxidant ferrostatin-1 (10 μM), the receptor-interacting serine/threonine-protein kinase 1 (RIPK1) inhibitor necrostatin-1 (30 μM), or the phosphoinositide 3-kinase (PI3K) inhibitor wortmannin (1 μM). The absorbance values were measured using the Tecan Infinite^®^ F200 plate reader (Männedorf, Switzerland). The percentage of LDH release was calculated by dividing the absorbance values by the maximum LDH release absorbance values. The results were derived from at least three independent experiments, each performed in triplicate wells.

### 2.6. Metabolic Activity Assay

The metabolic activity of the cells was evaluated using the Alamar Blue assay, which is based on the metabolic conversion of the non-fluorescent indicator dye resazurin to the fluorescent resorufin in living cells. The assay was performed according to the instructions provided by the manufacturer (Thermo Fisher Scientific, Uppsala, Sweden). After exposure to nanoparticles or to the soluble cobalt salt (Sigma), the supernatant was removed and 100 μL of a 1× solution of the Alamar Blue reagent in the complete medium was added to the cells. After 4 h of incubation at 37 °C, the fluorescence was recorded using the Tecan Infinite^®^ F200 plate reader. The results were derived from at least three independent experiments. The percentage of metabolic activity was quantified and expressed relative to the negative control (unexposed cells), which was set as 100%. 

### 2.7. Lipid Peroxidation Assay

The HT1080 and BJ cells were seeded in 6-well plates at a density of 2.5 × 10^5^ cells per well and incubated for one day prior to exposure. The following day, the cells were exposed to Fe_2_O_3_ or Fe_2_O_3_@Co-PEG nanoparticles for 24 h, with or without a 30 min pretreatment with the lipid antioxidant, Trolox (500 μM). To assess lipid peroxidation, the cells were incubated with a fluorescent probe, BODIPY 581/591 C11 (Thermo Fisher), at 5 μM for 30 min at 37 °C. Then, the cells were trypsinized and resuspended in 300 μL of Hanks’ balanced salt solution. The cell suspension was then strained through a 40 μm cell strainer and analyzed using a BD LSRFortessa™ X-20 flow cytometer equipped with a 488 nm laser; and the results were analyzed using the FlowJo™ version 10 software.

### 2.8. Western Blot Analysis

Western blotting was performed to evaluate the autophagy markers. Briefly, cell lysates were prepared according to the following protocol. First, cells were incubated in RIPA buffer [Tris-HCl (pH 7.4), NaCl (150 mM), NP40 (1%), Na-deoxycholate (0.25%), EDTA (1 mM), dithiothreitol (1 mM), and phenylmethylsulfonyl fluoride (1 mM)] with a freshly added protease inhibitor cocktail (Sigma Aldrich). Thereafter, the cell lysates were centrifuged at 12.000 rpm for 10 min and the resulting supernatants were collected for western blot analysis. The protein concentration was determined using the Pierce™ BCA Protein Assay Kit (Thermo Scientific, Waltham, MA, USA), and 30 μg of protein per sample was loaded onto a pre-cast SDS-PAGE 4–12% Bis-Tris gel (Thermo Fisher Scientific). The proteins were then transferred to PVDF membranes which were incubated overnight with a rabbit polyclonal anti-LC3B antibody (Abcam, Cambridge, UK) while a mouse anti-GAPDH antibody (Thermo Fisher) was used as a loading control. After washing, the membranes were incubated with fluorescently labeled anti-mouse or anti-rabbit secondary antibodies obtained from LI-COR Biosciences (Lincoln, NE, USA). The blots were scanned and imaged using the LI-COR Biosciences Odyssey^®^ imaging system. 

### 2.9. Statistical Analysis

The results were based on at least three independent experiments. The data were reported as mean values ± S.E.M. and the statistical analysis was conducted using the GraphPad Prism 5. One-way ANOVA was used to evaluate statistical significance, followed by either Dunnett’s or Tukey’s post hoc analysis. The level of statistical significance was established at *p* < 0.05.

## 3. Results

### 3.1. Cellular Internalization of Polymer-Coated Fe_2_O_3_ Nanoparticles

**Scheme 1:**
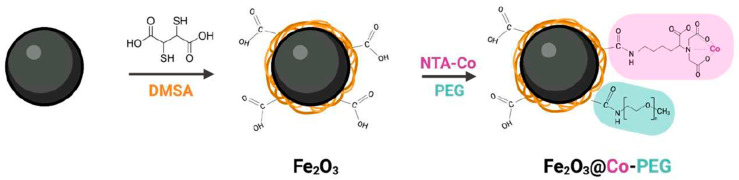
Functionalization of Fe_2_O_3_ nanoparticles. DMSA, dimercaptosuccinic acid; NTA, nitrilotriacetic acid; PEG, poly (ethylene glycol).

DMSA-coated Fe_2_O_3_ and Co-functionalized Fe_2_O_3_ nanoparticles were synthesized as described [15]. The functionalization of the nanoparticles is depicted in Scheme 1. Both nanoparticles were analyzed by TEM (Supplementary Figure S1) and DLS (Supplementary Figure S2) to verify the morphology of the particles and to establish the hydrodynamic diameter and ζ potential (in water). No significant differences could be observed between the Fe_2_O_3_ and Fe_2_O_3_@Co-PEG nanoparticles in terms of primary particle size (10.9 ± 2.7 nm and 10.7 ± 2.3 nm, respectively) (Supplementary Figure S1). An increase in the hydrodynamic diameter and a slight decrease in the ζ potential were noted for the Co-functionalized nanoparticles (Supplementary Figure S2). DLS was also performed in cell culture medium supplemented with serum; both nanoparticles were found to agglomerate (438.73 ± 21.45 nm and 352.93 ± 5.18 nm, respectively, for Fe_2_O_3_ and Fe_2_O_3_@Co-PEG). Moreover, the ζ potential values of the particles shifted to less negative values (−8.93 ± 0.90 mV and −7.77 ± 0.53 mV, respectively). This is commonly observed for nanoparticles dispersed in the presence of serum. 

**Figure 1:**
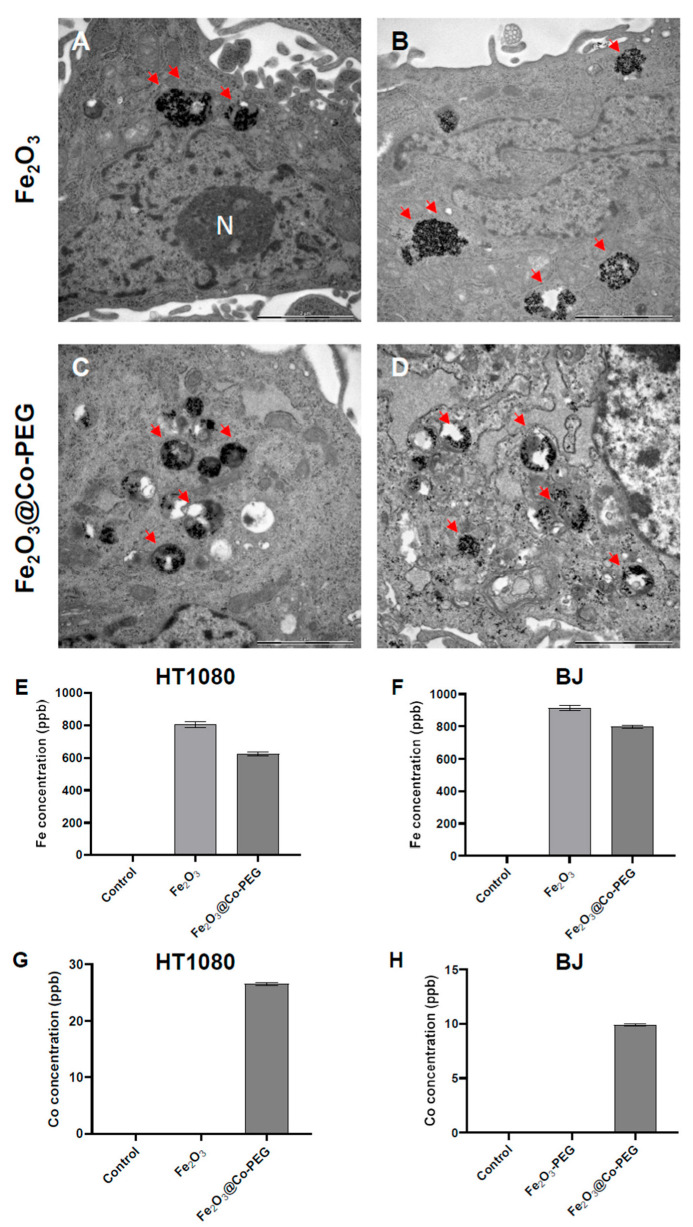
Cellular uptake of polymer-coated iron oxide nanoparticles. (**A**–**D**) TEM micrographs show cellular uptake in HT1080 cells (**A**,**C**) and BJ (fibroblast) cells (**B**,**D**) after 24 h of exposure to 50 μg/mL Fe_2_O_3_ nanoparticles (**A**,**B**) or Fe_2_O_3_@Co-PEG nanoparticles (**C**,**D**). The arrows indicate the presence of nanoparticles. N, cell nucleus. Scale bars in all panels: 2 μm. (**E**–**H**) ICP-MS measurements. Cellular concentration of Fe (**E**,**F**) and Co (**G**,**H**) in cells exposed to 50 μg/mL Fe_2_O_3_ or Fe_2_O_3_@Co-PEG for 24 h determined by ICP-MS.

We then investigated the uptake of the particles in cancer cells versus normal cells. HT1080 (fibrosarcoma) cells were selected because they are known to be susceptible to ferroptosis [21] while BJ cells represent their normal (human fibroblast) counterparts. Indeed, we confirmed that the HT1080 cell line is susceptible to the canonical ferroptosis activating agent, RSL3, which is believed to suppress GPX4 (Supplementary Figure S3). TEM analysis showed that the Fe_2_O_3_ nanoparticles were internalized by the HT1080 and BJ cells (Figure 1A,B). The Fe_2_O_3_@Co-PEG nanoparticles were also internalized in both cell lines (Figure 1C,D). The nanoparticles appeared to be clustered together in membrane-enclosed vesicles. Furthermore, the cellular content of Fe and Co was assessed by ICP-MS after exposure for 24 h to the Fe_2_O_3_ or Fe_2_O_3_@Co-PEG nanoparticles. The content of Fe in the HT1080 cells was significantly increased after exposure to Fe_2_O_3_ and Fe_2_O_3_@Co-PEG compared to untreated control (Figure 1E). In addition, the content of Fe in the HT1080 cells after exposure to Fe_2_O_3_@Co-PEG was slightly less than that of Fe_2_O_3_. Similar results were also seen in BJ (normal fibroblast) cells (Figure 1F). Moreover, cellular Co content was detected only in cells exposed to the cobalt-functionalized Fe_2_O_3_@Co-PEG nanoparticles, but not in cells exposed to the Fe_2_O_3_ or untreated control (Figure 1G,H). Interestingly, the cellular content of Co after the exposure of the BJ cells to Fe_2_O_3_@Co-PEG was considerably lower than that of the HT1080 cells (Figure 1G,H). This could be due to a lower rate of uptake or could be explained by the export of Co from the BJ cells. This remains to be studied in more detail, but our pilot studies showed that BJ cells express higher levels of ferroportin (*FPN1*) than HT1080 cells. Ferroportin is an iron exporter that is also reactive with Co [22]. Taken together, our results showed that the Fe_2_O_3_ and the Fe_2_O_3_@Co-PEG nanoparticles were taken up both in cancerous and normal cell lines.

### 3.2. Toxicity of Fe_2_O_3_ Nanoparticles with/without Cobalt Functionalization

**Figure 2:**
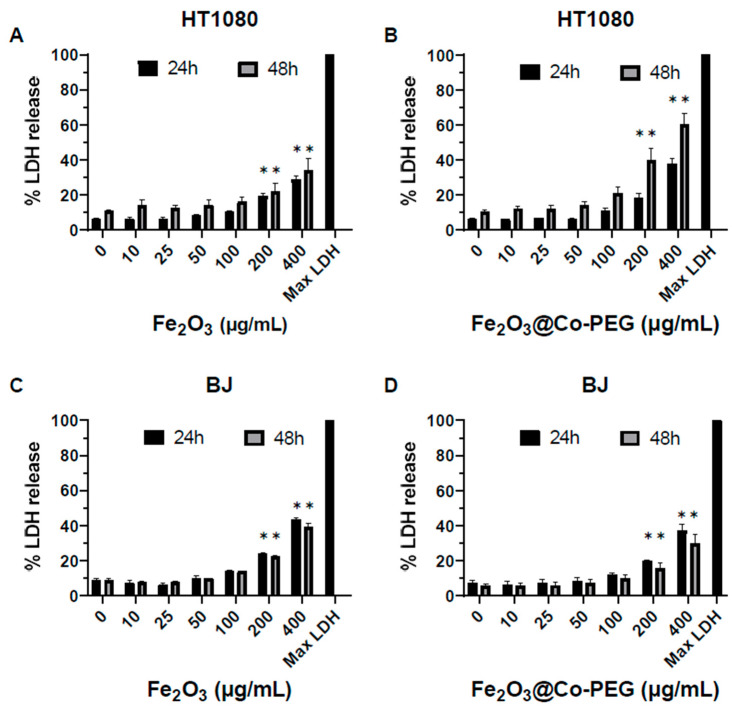
Cytotoxicity of polymer-coated iron oxide nanoparticles. The cytotoxicity towards HT1080 cells (**A**,**B**) and BJ cells (**C**,**D**), following exposure for 24 h or 48 h to the indicated concentrations of Fe_2_O_3_ nanoparticles (**A**,**C**) or Fe_2_O_3_@Co-PEG nanoparticles (**B**,**D**), was determined using the LDH release assay. Data shown are mean values ± S.E.M. of three independent experiments. * *p* < 0.05.

**Figure 3:**
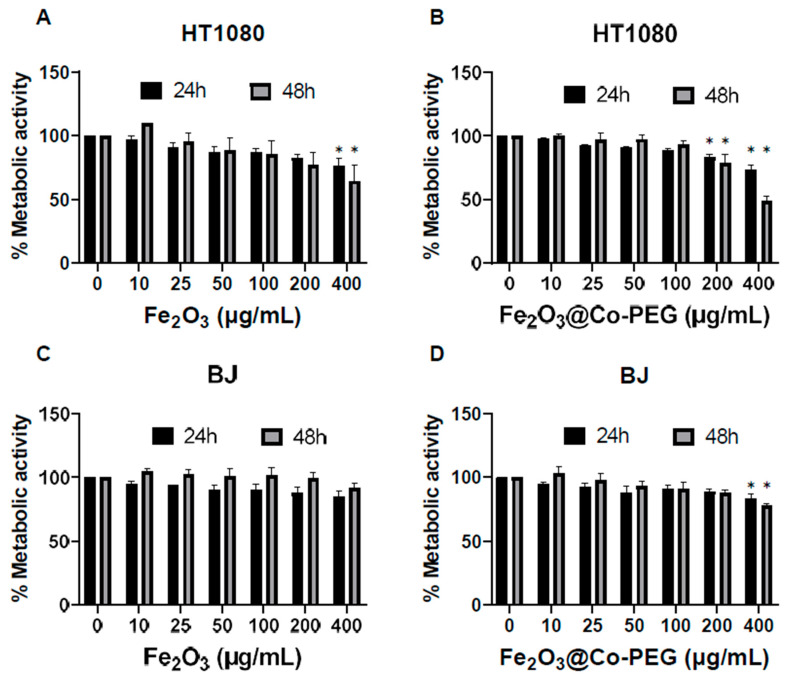
Impact of polymer-coated iron oxide nanoparticles on cellular metabolism. The Alamar Blue assay was used to measure the metabolic activity of HT1080 (**A**,**B**) and BJ cells (**C**,**D**) after exposure for 24 h and 48 h to the indicated concentrations of Fe_2_O_3_ nanoparticles (**A**,**C**) or Fe_2_O_3_@Co-PEG nanoparticles (**B**,**D**). Data shown are mean values ± S.E.M. of three independent experiments. * *p* < 0.05.

We then performed a cytotoxicity screening of Fe_2_O_3_ and Fe_2_O_3_@Co-PEG nanoparticles using the LDH release assay. No cytotoxicity was observed in the HT180 fibrosarcoma cells or BJ normal fibroblasts for both nanoparticles at concentrations up to 100 μg/mL (Figure 2A–D). However, at higher concentrations (200–400 μg/mL), cytotoxicity was observed; this was more pronounced for Fe_2_O_3_@Co-PEG as compared to Fe_2_O_3_ and more cytotoxicity was observed in HT1080 cells as compared to BJ cells (Figure 2A–D). To complement these results, we determined the metabolic activity of the two cell lines using the Alamar Blue assay, following exposure to the Fe_2_O_3_ and Fe_2_O_3_@Co-PEG nanoparticles (Figure 3A–D). The metabolic activity decreased more significantly in HT1080 cells at 24 h and 48 h than it did in BJ cells; this was observed for both nanoparticles. However, at the highest concentration, Fe_2_O_3_@Co-PEG appeared to be more cytotoxic compared to Fe_2_O_3_ for both cancer cells and fibroblasts (Figure 3A–D); thus, confirming the results obtained with the LDH release assay. We also investigated the cytotoxicity of soluble Co by exposing the cells to water-soluble Co (II) chloride (CoCl_2_). The metabolic activity of the HT1080 and BJ cells after exposure to CoCl_2_ was determined using the Alamar Blue assay. The results showed that (very) high concentrations of CoCl_2_ (100–200 μg/mL) significantly decreased the metabolic activity of HT1080 cells (Supplementary Figure S4A) and the effect was more pronounced at 48 h when compared to 24 h. The effect of CoCl_2_ was less pronounced in BJ cells; although, a dose-dependent effect was observed (Supplementary Figure S4B). 

### 3.3. High-Dose Exposure to Fe_2_O_3_ Nanoparticles Triggers Ferroptosis

**Figure 4:**
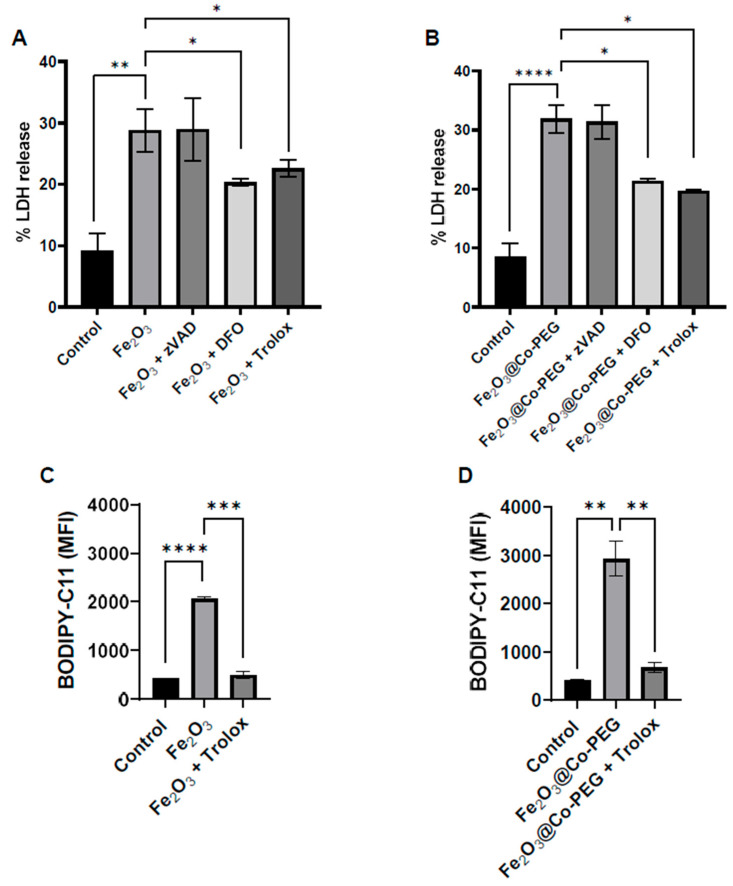
High-dose exposure to iron oxide nanoparticles triggers ferroptosis. The percentage of LDH release in HT1080 cells after exposure for 24 h to (**A**) Fe_2_O_3_ nanoparticles and (**B**) Fe_2_O_3_@Co-PEG nanoparticles at 400 μg/mL in the presence or absence of the specified inhibitors, zVAD-fmk (20 μM), DFO (10 μM), and Trolox (500 μM). Lipid peroxidation in cells exposed for 24 h to (**C**) Fe_2_O_3_ nanoparticles or (**D**) Fe_2_O_3_@Co-PEG nanoparticles at 400 μg/mL, in the presence and absence of Trolox (500 μM), was assessed using the flow cytometry-based C11-BODIPY 581/591 assay. The mean C11-BODIPY581/591 fluorescence intensity, reflective of the level of lipid peroxidation in the cells, is plotted. Data shown are the mean values ± S.E.M. of three independent experiments. * *p* < 0.05; ** *p* < 0.01; *** *p* < 0.001; **** *p* < 0.0001.

We then asked whether the maghemite nanoparticles triggered ferroptosis (at high concentrations). To this end, the two cell lines were exposed to the highest concentration (400 μg/mL) of Fe_2_O_3_ and Fe_2_O_3_@Co-PEG nanoparticles and cell death was monitored in the presence or absence of various cell death inhibitors. First, we noted that cell death was non-apoptotic as preincubation of the cells with the pan-caspase inhibitor zVAD-fmk failed to rescue the cells (Figure 4A,B). However, cells preincubated with the iron-chelating agent DFO or with the water-soluble vitamin E analog Trolox were significantly protected both from Fe_2_O_3_ and Fe_2_O_3_@Co-PEG nanoparticles (Figure 4A,B). Furthermore, lipid peroxidation was detected using the fluorescent probe BODIPY-C11. The results showed that Fe_2_O_3_ and Fe_2_O_3_@Co-PEG significantly increased the mean fluorescent intensity of BODIPY-C11 fluorescence when compared to the control, indicating the presence of lipid peroxides (Figure 4C,D). Moreover, Trolox completely reversed the lipid peroxidation induced by Fe_2_O_3_ and Fe_2_O_3_@Co-PEG (Figure 4C,D). Thus, these results showed that high concentrations of Fe_2_O_3_ or Fe_2_O_3_@Co-PEG triggered ferroptosis in the human HT1080 fibrosarcoma cell line. We also tested whether the cell death observed at high concentrations of these nanoparticles could be driven by other regulated necrosis pathways [23]. Specifically, we preincubated HT1080 cells with the necroptosis-inhibiting agent necrostatin-1. Interestingly, necrostatin-1 failed to rescue the cells exposed to Fe_2_O_3_ nanoparticles (Supplementary Figure S5A) while significant protection was observed in the case of Fe_2_O_3_@Co-PEG nanoparticles (Supplementary Figure S5B). Taken together, it appears that the Co-coated nanoparticles triggered mixed cell death (ferroptosis and necroptosis) in HT1080 cells.

### 3.4. Fe_2_O_3_ Nanoparticle-Triggered Ferroptosis Is Autophagy-Dependent

**Figure 5:**
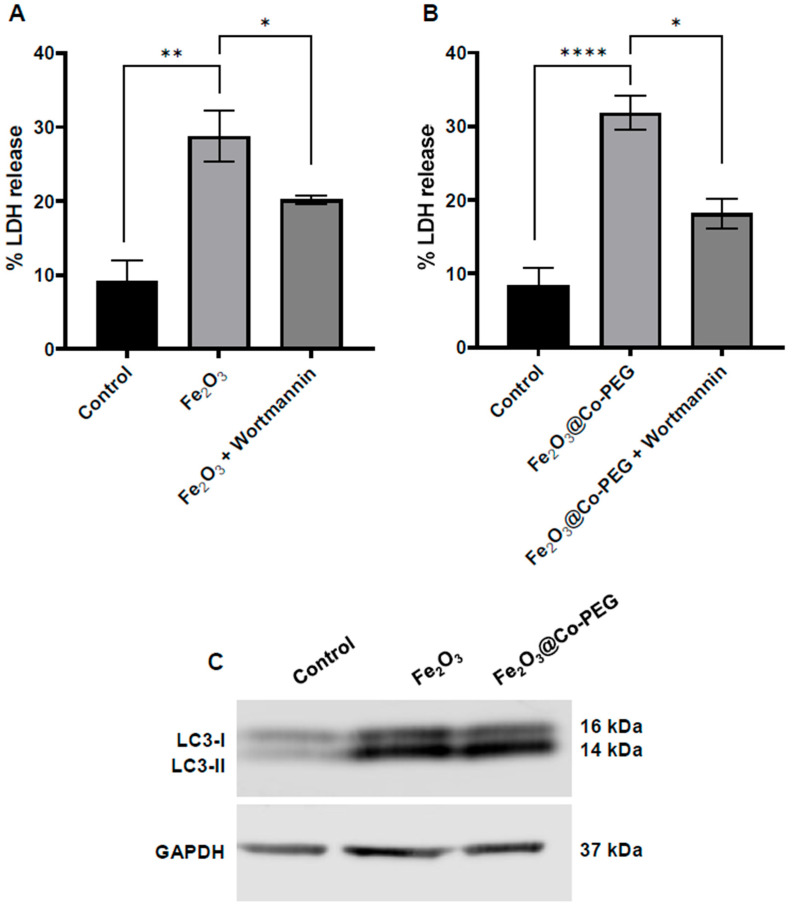
High-dose iron oxide nanoparticles trigger autophagy-dependent cell death. The percentage of LDH release in HT1080 cells after exposure for 24 h to (**A**) Fe_2_O_3_ nanoparticles and (**B**) Fe_2_O_3_@Co-PEG nanoparticles at 400 μg/mL in the presence or absence of the autophagy inhibitor, wortmannin (1 μM). Data shown are the mean values ± S.E.M. of three independent experiments. * *p* < 0.05; ** *p* < 0.01; **** *p* < 0.0001. The values for the control samples and nanoparticles alone in panels (**A**,**B**) are identical to the values shown in Figure 4A,B. (**C**) The expression of LC3-I and LC3-II was investigated by Western blot in HT1080 cells following exposure for 24 h to Fe_2_O_3_ and Fe_2_O_3_@Co-PEG nanoparticles at 400 μg/mL. GAPDH was used as a loading control.

It has been suggested that ferroptosis is an autophagy-dependent form of cell death [24]. To address this, we preincubated HT1080 cells with wortmannin, a known autophagy inhibitor, and determined its impact on cell death induced by the maghemite nanoparticles at the highest concentration (400 μg/mL). Preincubation with wortmannin significantly reduced cell death triggered by Fe_2_O_3_ and Fe_2_O_3_@Co-PEG nanoparticles (Figure 5A,B). The lipidation of microtubule-associated light chain-3 (LC3-I) to form LC3-II is an essential step in autophagosome formation. To obtain further evidence for the involvement of autophagy, we performed Western blot analysis for LC3. Both Fe_2_O_3_ and Fe_2_O_3_@Co-PEG nanoparticles increased the expression of LC3I/II compared to the control, and the LC3II band was disproportionally induced for both nanoparticles (Figure 5C). Altogether, our results suggested that Fe_2_O_3_ and Fe_2_O_3_@Co-PEG induced autophagy-dependent ferroptosis in the HT108 cell line.

### 3.5. No Cytotoxicity Elicited by PEG-PLGA-Modified Fe_3_O_4_ Nanoparticles

Finally, we asked whether Fe_3_O_4_-PEG-PLGA nanoparticles displayed any cytotoxicity. These nanoparticles are envisioned for a range of applications, including as a magnetic hyperthermia agent, as an imaging (contrast) agent, and for drug delivery [25]. For these reasons, we decided to complement our experiments in the HT1080 cell line with two additional cell lines representative of the innate immune system. THP-1 cells (differentiated into macrophage-like cells) and HL-60 cells (differentiated into neutrophil-like cells) were thus included. The nanoparticles were characterized with respect to the physicochemical properties in a previous study [18]. The primary particle size (by TEM) was 12 ± 4 nm. Fe_3_O_4_-PEG-PLGA displayed excellent stability in a cell culture medium supplemented with 10% FBS (hydrodynamic diameter 114.60 ± 0.65 nm by DLS) but were found to sediment in cell medium in the absence of serum (Supplementary Figure S6). Furthermore, using the LAL assay [26], the particles were shown to be endotoxin-free.

The cells were exposed to Fe_3_O_4_-PEG-PLGA nanoparticles for 24 h and cell viability was monitored using the LDH release assay. No cytotoxicity was observed in HT1080, THP-1, or HL-60 cells up to 100 μg/mL (higher concentrations could not be achieved) (Supplementary Figure S7A–C).

## 4. Discussion

The present study has shown that the polymer-coated iron oxide nanoparticles tested were largely non-cytotoxic toward cancer cells and normal fibroblasts; however, high-dose exposure triggered autophagy-dependent ferroptosis. Ferroptosis is a “novel” cell death mechanism that appears to be especially relevant for cancer cells [3]. The iron oxide nanoparticles functionalized with Co were found to be somewhat more cytotoxic (especially towards HT1080 cells) and we provided some evidence for a mixed cell death involving ferroptosis and necroptosis. The observation that high concentrations of the Co-functionalized iron oxide nanoparticles triggered cell death with features of both ferroptosis and necroptosis (but not apoptosis) is a novel one that deserves further attention. Emerging evidence suggests the existence of molecular crosstalk between the two modes of cell death, suggesting that cancer cells are not always “monotheistic” (in other words, more than one cell death modality may co-exist, at least in cancerous cells) [27,28]. However, the current effects were observed at 400 μg/mL and cannot be taken as a sign that Co-coated nanoparticles are candidates for cancer therapy as such concentrations may be difficult to achieve in vivo, and because cell death was noted in normal BJ cells (at high concentrations). 

Previous work has shown that CoCl_2_ triggered RIPK3 and MLKL-dependent cell death (necroptosis) in mouse fibroblast cells lines (NIH/3T3 and L929); however, RIPK1 appeared to be less involved in the latter model as necrostatin-1 only provided a modest protective effect [29]. On the other hand, we previously found that Co-based nanoparticles as well as CoCl_2_ triggered ferroptosis-like cell death in neuronal cells [20]. Thus, there may well be cell type-dependent differences in terms of the sensitivity and cellular responses to Co. Nevertheless, the present results suggest that Co-functionalized iron oxide nanoparticles may engage more than one cell death modality (if administered at high concentrations). It is worth noting that the Co ions were introduced by taking advantage of the chelating agent NTA (Scheme 1); this Co-NTA coordination complex is widely used to isolate and purify recombinant His-tagged proteins. Previous studies have shown that NTA-Co binding remains stable for up to 24 h in cell culture medium [30]. However, a decrease in pH will lead to the dissociation of the bound metal. Thus, when nanoparticles are internalized by cells and trafficked to the endo-lysosomal compartment, the Co could be released from the particles. On the other hand, our studies using soluble CoCl_2_ as a reference have shown that very high concentrations of Co are required to drive cell death in HT1080 cells. Indeed, it is important to consider the fact that we have observed cell death for Fe_2_O_3_ nanoparticles (with or without Co) only at very high concentrations (200–400 μg/mL); meanwhile, other investigators recently reported that gallic acid-coated iron oxide nanoparticles elicited ferroptosis in the same cell line (HT1080) at a much lower concentration (the half-maximal effective concentration was reported to be 3 μg/mL in the latter study) [6]. However, the surface coating could play a key role, as we have shown previously for Cu-based nanoparticles [31]. Notably, other investigators have reported that gallic acid, a natural phenolic compound with known anti-cancer properties, triggered cell death in the cervical carcinoma cell line HeLa with features of apoptosis, necroptosis, and ferroptosis [32]. Importantly, the gallic acid-induced cell death was completely abrogated by DFO. Thus, when different iron oxide nanoparticles are studied and compared, the surface coating (as well as the presence of other potentially cytotoxic entities, such as Co) needs to be taken into account. Zhang et al. [10] reported that DMSA-coated Fe_3_O_4_ and Fe_2_O_3_ nanoparticles triggered ferroptosis (albeit to a very modest degree) in endothelial cells while polyglucose sorbitol carboxymethyether (PSC)-coated Fe_2_O_3_ failed to do so; the authors argued that these differences could be explained by differences in the uptake of DMSA versus PSC-coated particles. In the present study, however, Fe_2_O_3_ and Fe_2_O_3_@Co-PEG nanoparticles were both stabilized with DMSA, which did not prevent cellular uptake. To summarize, while surface coatings, including PEG, may certainly affect nanoparticle uptake [33,34], the capping agents could also be directly involved in the induction of cell death, as shown for gallic acid (see above). 

The core chemistry, specifically, the valence state of iron-based nanomaterials, may also play a role in their ferroptosis potential [35]. Furthermore, the availability of Fe in the cells is important. Ferroptosis is a form of cell death that is induced by iron-dependent lipid peroxidation in the face of compromised defense mechanisms for the elimination of oxidized lipids [36]. However, it is an oversimplification to suggest that the cellular uptake of iron-based nanomaterials is sufficient for driving ferroptosis. Iron is subject to strict control in (cancer) cells, and iron import, export, storage, and turnover impact ferroptosis sensitivity [37]. We have shown here, using ICP-MS, that the cellular iron content was greatly increased in cancer cells (HT1080) and normal fibroblasts (BJ) exposed to polymer-coated iron oxide nanoparticles; we also demonstrated that the iron-chelating agent DFO blocked cell death in HT1080 cells following exposure to high concentrations of Fe_2_O_3_ and Fe_2_O_3_@Co-PEG nanoparticles. However, further studies are required to understand the fate and turnover of iron in cells subjected to iron oxide nanoparticles. Gao et al. [8] used X-ray absorption near-edge structure (XANES) for the speciation of iron in human breast cancer cells exposed to ultrasmall iron oxide nanoparticles. They provided evidence that iron existed as Fe^2+^ at the early time points (up to 12 h); during this period, the Fenton reaction occurred, leading to lipid peroxidation. However, Fe^2+^ was undetectable at 48 h and the particles were transformed into ferritin following long-term exposure (72 h). Ferritin is a major intracellular iron storage protein complex and increased ferritin limits ferroptosis. It is notable that oncogenic *RAS*-harboring cancer cells (such as HT1080) display increased iron content relative to their normal cell counterparts due to an upregulation of transferrin receptor 1 and downregulation of ferritin heavy chain 1 and ferritin light chain [38]. This may explain why these cells are more susceptible to RSL3 (an inhibitor of GPX4) and perhaps also why they are more sensitive to iron-based nanomaterials. However, as we have shown in the present study, normal fibroblasts (BJ) are also sensitive to high-dose exposure to iron oxide nanoparticles, albeit to a lesser extent than HT1080. 

We also provided evidence that iron oxide nanoparticles triggered autophagy-dependent ferroptosis in HT1080 cells, in accordance with other recent studies of iron oxide nanoparticles [9]. Khan et al. [39] found that iron oxide nanoparticles triggered autophagy in lung adenocarcinoma cells, but not in normal cells (fibroblasts), and the pretreatment of cancer cells with autophagy inhibitors promoted cell viability. Other investigators have demonstrated that the surface coating of iron oxide nanoparticles may dictate intracellular trafficking and autophagy induction in cancer cells and normal cells [40,41]. As discussed above, ferroptosis is believed to be an autophagy-dependent form of cell death [24]. The connections between autophagy and ferroptosis in cells exposed to nanoparticles are multifaceted. Thus, while it is possible that the endocytosis of iron oxide nanoparticles leads to ferroptosis by increasing intracellular levels of labile iron, the autophagic degradation of ferritin has also been shown to trigger ferroptosis by increasing the labile iron pool [42]; this possibility cannot be excluded. Notably, the autophagic turnover of ferritin (ferritinophagy) is dependent on the delivery of ferritin to the lysosomes [43]; a recent study has shown that the autophagy induction by ZnO nanoparticles requires ferritinophagy [44]. 

Autophagy is defined as a catabolic process that delivers cytoplasmic components and organelles to the lysosomes for digestion (“self-eating”). Moreover, endocytosis is the main route by which nanoparticles enter cells, meaning that nanoparticles (including iron oxide nanoparticles) are trafficked to the endo-lysosomal compartment [5]. Thus, the lysosomes sit at the nexus of iron metabolism, ferroptosis, and autophagy, with these organelles also playing a key role in the cellular trafficking of nanoparticles in most, if not all, cells [45].

## 5. Conclusions

Here, we showed that polymer-coated iron oxide nanoparticles are readily taken up by human cancer cells (fibrosarcoma) and normal fibroblasts; however, no cytotoxicity was observed at concentrations up to 100 μg/mL. On the other hand, high-dose exposure to maghemite (Fe_2_O_3_) nanoparticles triggered ferroptosis with signs of autophagy in cancer cells. Overall, not all iron oxide nanoparticles are alike and the induction of cell death (ferroptosis) in cancer cells must be considered in light of adverse effects on normal cells.

## Data Availability

The original data reported in this study are available from the corresponding author on reasonable request.

## Supplementary Materials

The following supporting information can be downloaded at: https://www.mdpi.com/article/10.3390/nano13111719/s1, Figure S1. Characterization of Fe2O3 nanoparticles. Figure S2. Characterization of Fe2O3 nanoparticles. Figure S3. HT1080 cells are susceptible to ferroptosis. Figure S4. Cobalt chloride (CoCl2)-induced cytotoxicity. Figure S5. Co-doped nanoparticles trigger necroptosis. Figure S6. Characterization of Fe3O4 -PEG-PLGA particles. Figure S7. Fe_3_O_4_ -PEG-PLGA particles are non-cytotoxic.
