# Supplementary material for: High-Dose Exposure to Polymer-Coated Iron Oxide Nanoparticles Elicits Autophagy-Dependent Ferroptosis in Susceptible Cancer Cells
Source: Nanomaterials (Basel). 2023 May 24;13(11):1719. doi: 10.3390/nano13111719 (PMC10254322; doi:10.3390/nano13111719)
Supplement: Supplementary file 1 [file nanomaterials-13-01719-s001.zip › nanomaterials-2378377-supplementary.pdf]

Supporting information

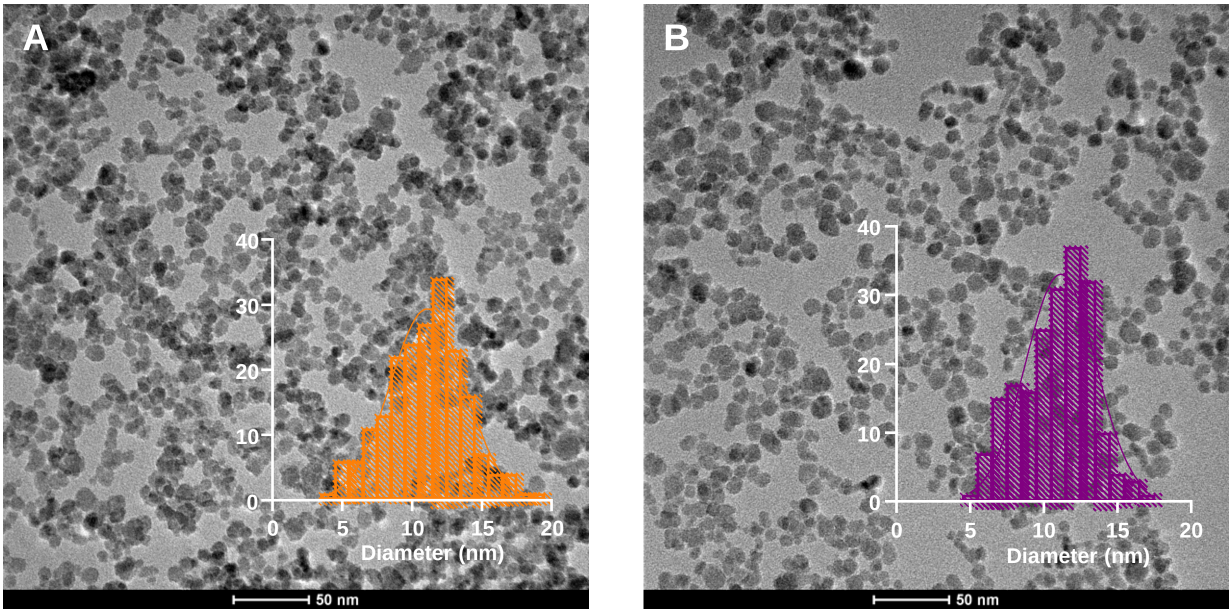

Figure S1. Characterization of Fe_2O_3 nanoparticles. TEM micrographs of (A) Fe_2O_3 and (B) Fe_2O_3-Co-PEG. The size distribution was obtained by counting at least 100 NPs. Scale bars: 50 nm.

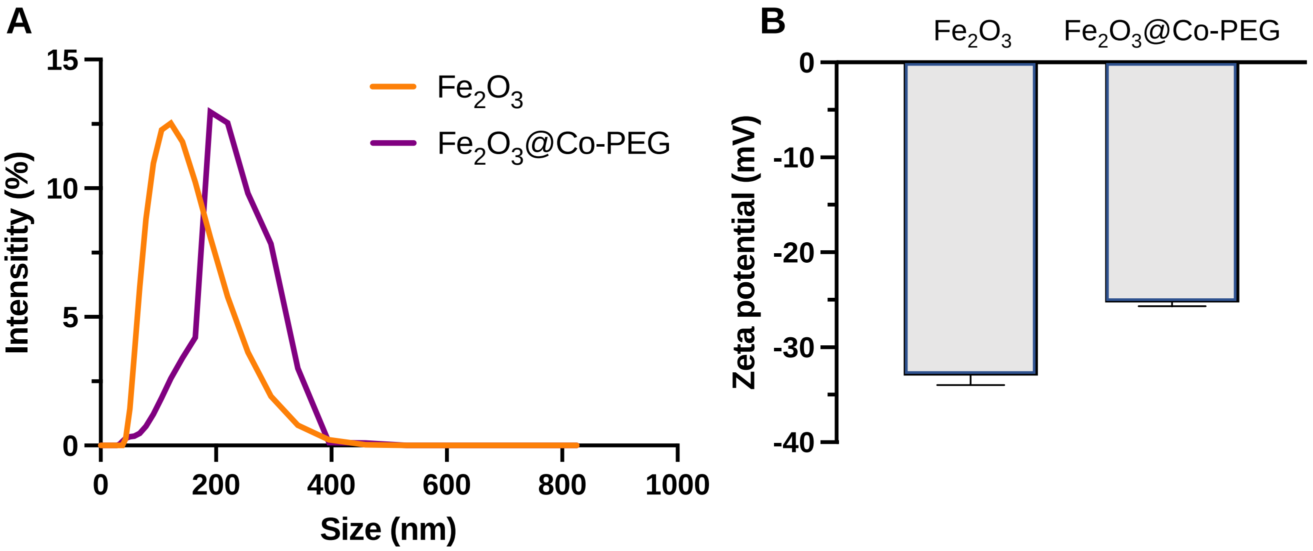

Figure S2. Characterization of Fe_2O_3 nanoparticles. Hydrodynamic diameter (A) and zeta potential (B) of Fe_2O_3 and Fe_2O_3-Co-PEG diluted in Milli-Q® water at 20 µg Fe/mL. The measurements were performed by dynamic light scattering (DLS) using a Malvern Zetasizer Nano ZS.

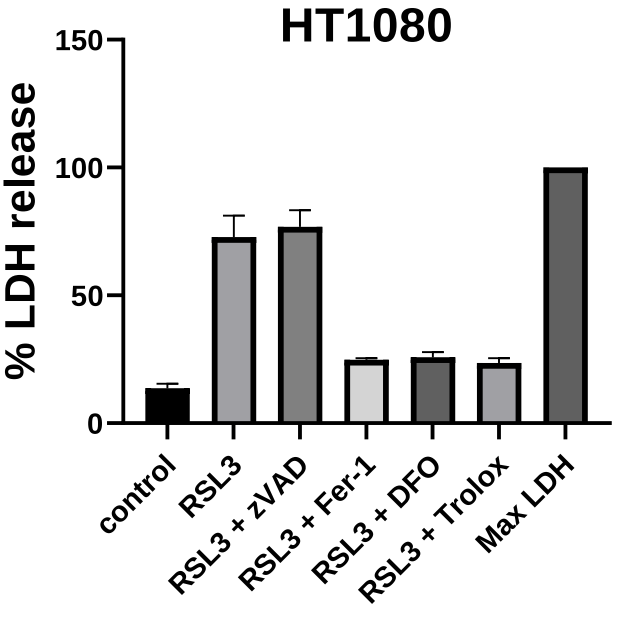

Figure S3. HT1080 cells are susceptible to ferroptosis. HT1080 cells were exposed to RSL3 (0.5 µM) for 24 h and cell death was monitored using the LDH release assay. The pan-caspase inhibitor zVAD-fmk failed to rescue the cells indicating that cell death was non-apoptotic. On the other hand, ferrostatin-1 (antioxidant), DFO (iron-chelating agent), and Trolox (vitamin E analog) all blocked RSL3-induced cell death in this model. Data shown are mean values ± S.E.M. (n=3).

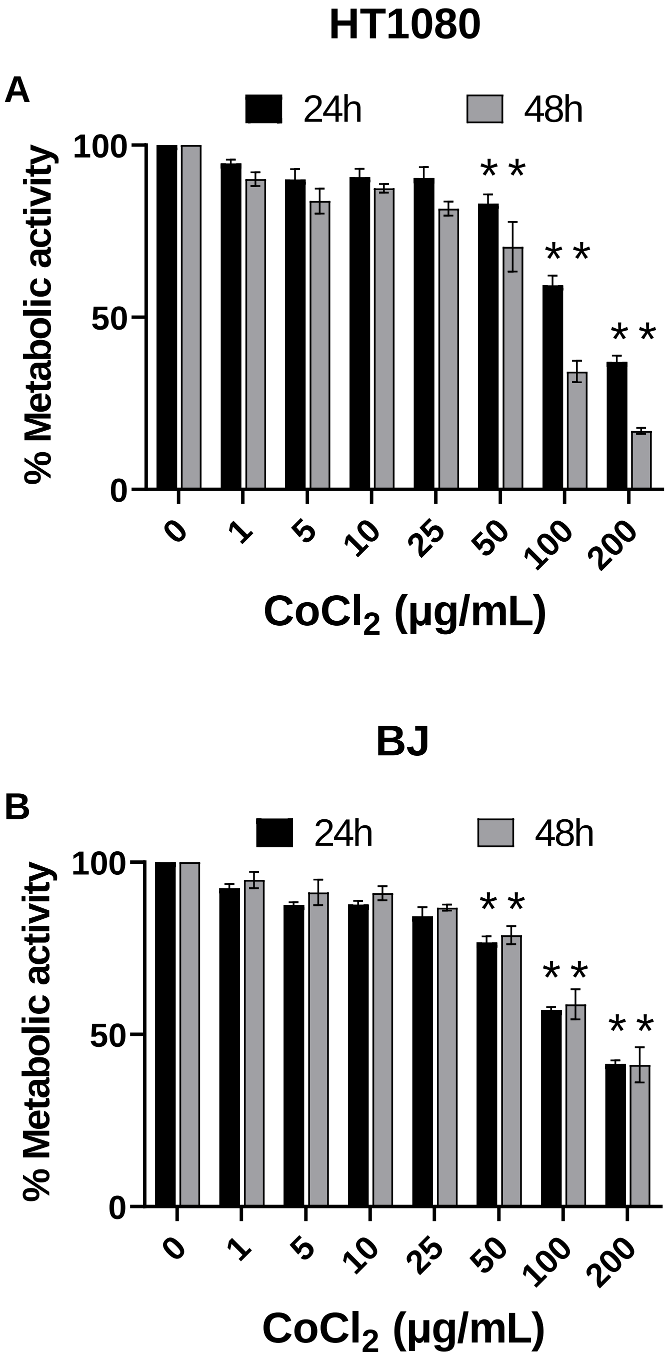

Figure S4. Cobalt chloride ($CoCl_2$)-induced cytotoxicity. The Alamar Blue assay was used to determine the cytotoxicity of $CoCl_2$ towards (A) HT1080 cells and (B) BJ cells at 24 h and 48 h. The LDH release assay could not be used due to assay interference with $CoCl_2$ (data not shown). The data are shown as mean values ± S.E.M. of three independent experiments. *$P < 0.05$.

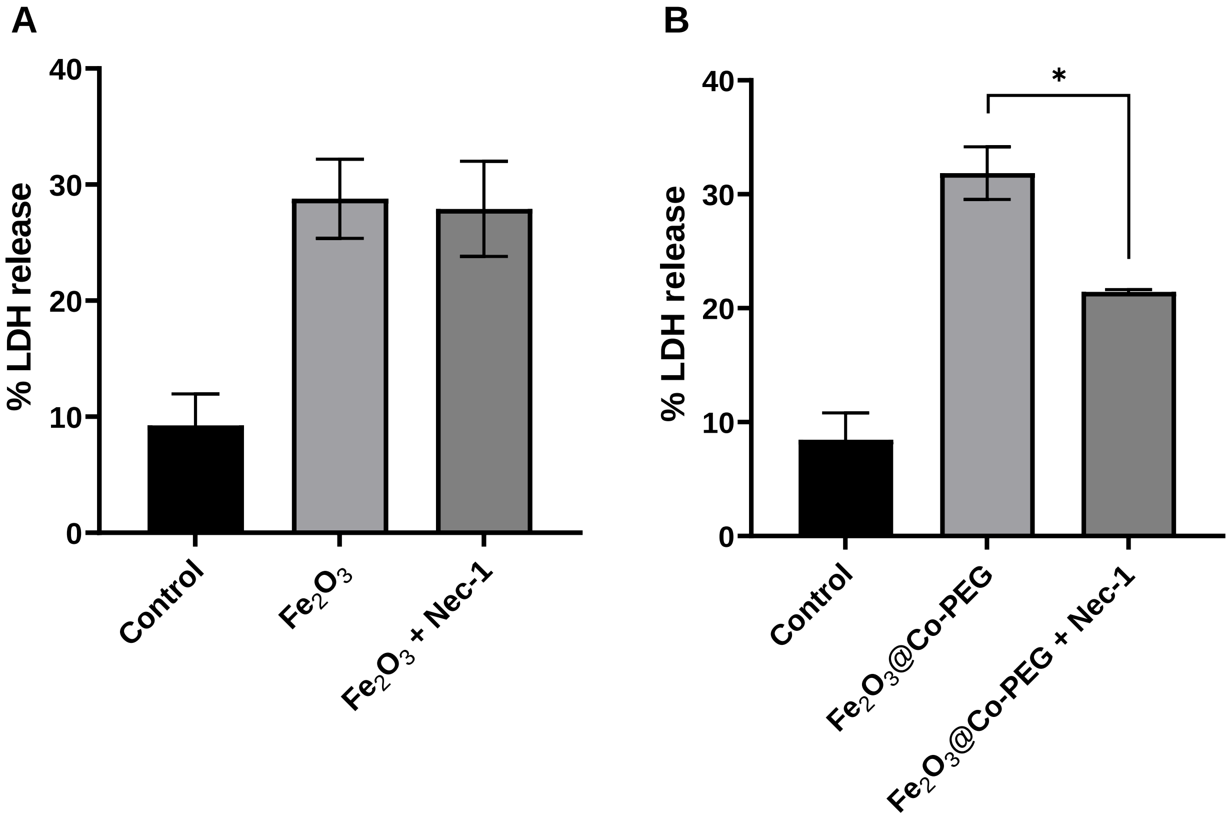

Figure S5. Evidence for necroptosis in cells exposed to Co-doped nanoparticles. HT1080 cells were exposed to a high concentration (400 µg/mL) of (A) Fe_2O_3 nanoparticles and (B) Fe_2O_3@Co-PEG nanoparticles for 24 h with and without the pre-incubation for 30 min with the RIPKI inhibitor, necrostatin-1 (30 µM). The values for the control samples and nanoparticles alone in panel A and B are identical to the values shown in Figure 4A and B. The data are shown as mean values ± S.E.M. of three independent experiments. *$P < 0.05$.

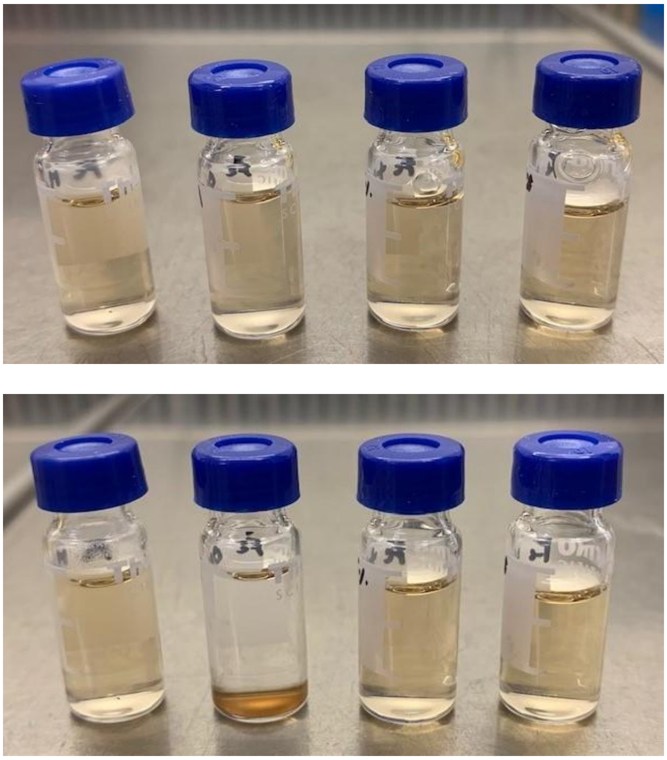

Figure S6. Characterization of Fe_3O_4-PEG-PLGA nanoparticles. The nanoparticles were diluted at 25 µg/mL in water or in cell culture medium (CCM) supplemented with 5 or 10% fetal bovine serum (FBS) and photographs were taken at 0 h (top row) and at 24 h (bottom row) to provide visual evidence of the stability of the dispersions. Samples from left to right: Fe_3O_4-PEG-PLGA in H_2O, CCM, CCM+5% FBS, CCM+10% FBS.

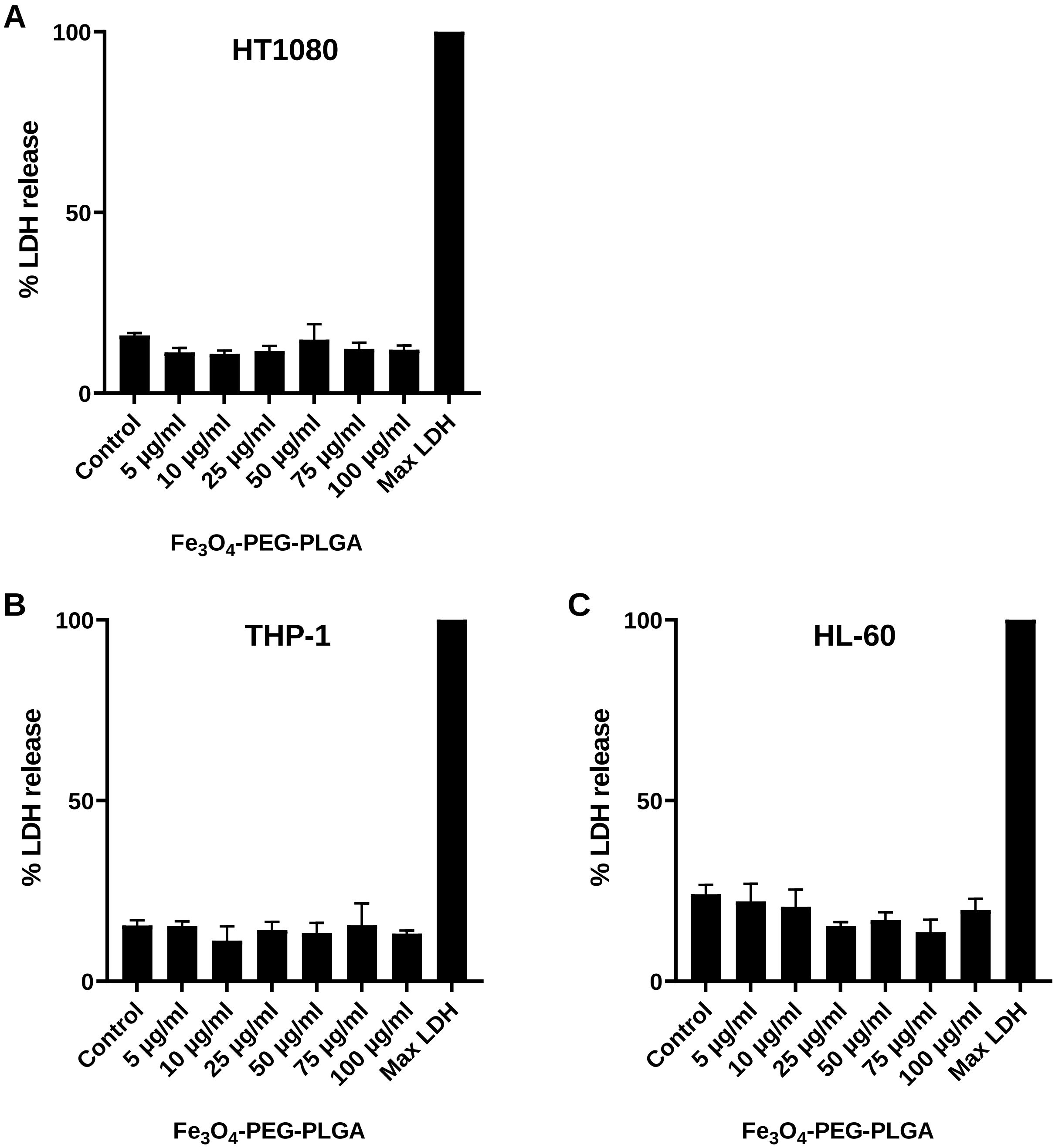

Figure S7. No cytotoxicity observed for Fe_3O_4-PEG-PLGA nanoparticles. The potential cytotoxicity was investigated using (A) HT1080 fibrosarcoma cells, (B) macrophage-differentited THP-1 cells, and (C) neutrophil-differentiated HL-60 cells. Cells were exposed to the indicated concentrations for 24 h and cytotoxicity was determined using the LDH release assay. The data are shown as mean values ± S.D. of three independent experiments. *$P < 0.05$.
